# Cytosine methylation at CpCpG sites triggers accumulation of non-CpG methylation in gene bodies

**DOI:** 10.1093/nar/gkw1330

**Published:** 2017-01-04

**Authors:** Nicolae Radu Zabet, Marco Catoni, Filippo Prischi, Jerzy Paszkowski

**Affiliations:** 1 The Sainsbury Laboratory, University of Cambridge, Cambridge, CB2 1LR, UK; 2 School of Biological Sciences, University of Essex, Colchester, CO4 3SQ, UK

## Abstract

Methylation of cytosine is an epigenetic mark involved in the regulation of transcription, usually associated with transcriptional repression. In mammals, methylated cytosines are found predominantly in CpGs but in plants non-CpG methylation (in the CpHpG or CpHpH contexts, where H is A, C or T) is also present and is associated with the transcriptional silencing of transposable elements. In addition, CpG methylation is found in coding regions of active genes. In the absence of the demethylase of lysine 9 of histone 3 (IBM1), a subset of body-methylated genes acquires non-CpG methylation. This was shown to alter their expression and affect plant development. It is not clear why only certain body-methylated genes gain non-CpG methylation in the absence of IBM1 and others do not. Here we describe a link between CpG methylation and the establishment of methylation in the CpHpG context that explains the two classes of body-methylated genes. We provide evidence that external cytosines of CpCpG sites can only be methylated when internal cytosines are methylated. CpCpG sites methylated in both cytosines promote spreading of methylation in the CpHpG context in genes protected by IBM1. In contrast, CpCpG sites remain unmethylated in IBM1-independent genes and do not promote spread of CpHpG methylation.

## INTRODUCTION

DNA methylation is a heritable epigenetic mark that affects gene regulation, mostly at the transcriptional level (1,2). In mammals, DNA is methylated predominantly at cytosines in the CpG sequence context, whilst in plants methylation in non-CpG sequences (CpHpG and CpHpH, where H can be A, C or T) is also present and contributes to epigenetic regulation (3).

In *Arabidopsis thaliana*, METHYLTRANSFERASE 1 (MET1) is the main methyltransferase active in the inheritance of CpG methylation during DNA replication (4,5). In the maintenance mechanism of CpG methylation, it is assumed that MET1 recognizes hemimethylated CpG sites and adds methylation to the unmethylated newly synthesized DNA strand.

Methylation in the CpHpG context is maintained through a positive feedback loop in which KRYPTONITE (KYP; also known as SUVH4), SUVH5 and SUVH6 recognize CpHpG methylation and add two methyl groups to lysine 9 of histone 3 (H3K9me2) (6). This mark is then recognized by CHROMOMETHYLASE 3 (CMT3) or CHROMOMETHYLASE 2 (CMT2), which results in the methylation of unmethylated cytosines in the CpHpG or CpHpH contexts, respectively (7).

Methylation in the CpHpH context is maintained by the RdDM pathway (RNA-directed DNA methylation), where 24-nt small interfering RNAs (siRNAs) synthesized by the synchronized activities of RNA POLYMERASE IV (Pol IV), RNA-DIRECTED RNA POLYMERASE 2 (RDR2) and DICER-LIKE 3 (DCL3) target DOMAIN REARRANGED METHYTRANSFERASE 2 (DRM2) to corresponding loci (3,8). The targeting step includes loading of siRNAs onto ARGONAUTE 4 (AGO4). Binding of AGO4 to Pol V (or its transcripts) recruits DRM2, which then methylates the DNA homologous to the siRNAs. In fact, this process is assumed to be the main mechanism of *de novo* methylation of cytosine in all sequence contexts.

Whilst the mechanism propagating CpG methylation seems to be well defined, maintenance of the DNA methylation patterns in non-CpGs is less clear. Especially how and to what degree the three pathways interact is not well understood, although certain connections have been observed. For example, CpHpG methylation seems to depend also on the RdDM pathway and there are two classes of CpHpG sites: (i) those that are targets of CMT3 (and to a much lower extent of CMT2) and (ii) those sites that, in addition to CMT2/3, are also targeted by the RdDM pathway (9,10). Furthermore, CMT2 can methylate DNA in both CpHpG and CpHpH contexts, which may contribute to the co-existence of the two modes of methylation at many chromosomal targets, mostly transposons (10). In addition, KRYPTONITE/SUVH4 (KYP) was shown to bind with a similar affinity to DNA methylated in both CpHpG and CpHpH contexts (6,11), explaining why regions that display CpHpG methylation may also acquire CpHpH methylation. Moreover, PolV seems to be recruited at a subset of CpG methylated *loci* (12), which suggests that CpG methylation is also involved in the maintenance of CpHpH methylation.

All types of methylation are found at transcriptionally silent transposons and their remnants. In addition, CpG methylation alone is present in coding regions of active genes. Genes containing this type of methylation are protected from an invasion of non-CpG methylation, especially CpHpG methylation (13). For a subset of body-methylated genes this is achieved by the demethylase of H3K9me2 (INCREASE IN BONSAI METHYLATION1—IBM1), which interferes with the self-reinforcing regulatory loop of CMT3/KYP (14). Nevertheless, there are body-methylated genes that, despite the depletion of IBM1 in *ibm1* mutants, are not invaded by CpHpG methylation. The cause of the resistance of this class of genes to CpHpG methylation is not clear.

Recently, it was observed that a *met1* mutation in *Physcomitrella patens* results in drastic depletion of methylation in ^m^CpCpGs but not ^m^CpApGs or ^m^CpTpGs (15). Further analysis of the available methylation dataset of the *A. thaliana met1-6* mutant (16) revealed a similar rule (15). These important results suggested the involvement of MET1 in the maintenance of methylation at CpHpG sites and a model has been proposed for the cooperation between MET1 and CMT3 in the maintenance of double methylation in CpCpGs at heavily methylated transposons; however, the role of MET1-mediated methylation of the internal cytosine at CpCpGs in directing *de novo* CpHpG methylation has not been addressed.

Whilst studying differential spreading of CpHpG in gene body-methylated genes, we have now found a possible explanation for the establishment and inheritance of aberrant CpHpG methylation at these DNA methylation targets. More specifically, we have found that CMT3 can methylate CpCpG sites only when the internal cytosines (which are in the CpG context) are methylated. Therefore, the body of methylated genes, in which methylation of internal cytosines of CpCpG sites is absent, are ‘epigenetically protected’ against invasion of CpHpG methylation in an IBM1-independent fashion.

## MATERIALS AND METHODS

### Bisulfite sequencing datasets

We used bisulfite sequencing datasets of wild-type (WT) and nine epigenetic mutants of *A. thaliana* in the Columbia background: WT (GSM1242401 and GSM980986), first generation *met1-3* (GSM981031), *drm1/2 cmt2/3* (*ddcc)* (GSM1242404), *cmt3* (GSM981003), *cmt2* (GSM981002), *cmt2/3* (GSM1242402), *suvh4* (GSM981057), *suvh4/5/6* (GSM981060), *drm1/2* (GSM981015) and second generation *ibm1* (GSM981026). The dataset was generated using leaves from 3-week-old plants grown under continuous light (9,10). We pooled the reads from the two biological replicates of WT plants. We considered all cytosines in the ^m^CpG (852 905), ^m^CpApG (167 958), ^m^CpTpG (155 869), ^m^CpCpG (60 239; where we considered the methylation level of the external cytosine only) and ^m^CpHpH (412 402) contexts that produced at least five reads in all samples and displayed at least 50% methylation in the CpG context and 25% in the non-CpG context in WT plants.

For *met1-1* and MET1 transgenic lines, we used the bisulfite sequencing dataset given in (Catoni *et al.*, bioRxiv: http://biorxiv.org/content/early/2016/06/08/057794). This dataset was generated using pools of 2-week-old seedlings (25–30 plants per pool) grown under long-day conditions (21°C, 16 h light, 8 h dark). The *met1-1* plants used were 13th generation homozygous *met1-1* derived from *A. thaliana* Columbia-0. For our analysis, we pooled the reads of the bisulfite sequencing datasets for the two MET1 transgenic lines.

To analyze the bisulfite sequencing datasets, we used Bismark tool (17) with bowtie2 (18) and computed the methylation percentage of each cytosine. The scripts used to process the data were deposited at https://github.com/nrzabet/A_thaliana_epigenetic_mutants.

### Generated datasets

Samples for bisulfite sequencing were obtained from 3-week-old rosettes of first generation *ibm1-1* homozygous mutant plants in the Col-0 and Ler-0 ecotypes (14) grown under long-day conditions (21°C, 16 h light, 8 h dark). The Ler-0 *ibm1-1* mutant, kindly provided by Dr H. Saze, was obtained after four backcrosses to the Ler-0 genotype starting from the Col-0 mutant line. DNA was extracted using the DNeasy Plant Mini Kit (Qiagen) following the manufacturer's instructions. DNA bisulfite conversion was performed starting from 150 ng of genomic DNA using the EZ-DNA Methylation-Gold Kit (Zymo Research) followed by DNA library preparation with the TruSeq DNA methylation Kit (Illumina) according to the manufacturer's instructions. The library quality and fragment sizes were controlled with a TapeStation 2200 (Agilent) instrument and the DNA quantified by PCR on a LightCycler 480 II (Roche) using the Library Quantification Kit (Kapa Biosystem). The DNA libraries were pooled at a concentration of 4 nM and sequenced with 2 × 75-bp paired-end reads on an Illumina NextSeq 500 instrument. Sequences reads were aligned using Bismark (17) against the *A. thaliana* genome TAIR10 version and the PacBio Ler-0 genome assembly (http://www.pacb.com/uncategorized/new-data-release-arabidopsis-assembly/) for Col-0 and Ler-0 *ibm1* mutants, respectively. Duplicated reads were collapsed into one read. Chloroplast sequences were used to estimate the bisulfite conversion.

We partitioned the reference genome of Col-0 (TAIR10) into 500-bp tiles and selected all tiles in WT displaying a mean gene body CpG methylation in 500 bp of at least 10% and non-CpG methylation lower than 5% (47 376 bins). We then used BLAT (19) to map these regions to the Ler-0 genome assembly. We kept only bins with an alignment of 400–600 bp and at least one CpCpG site (31 994 bins). Since the *ibm1* mutation does not affect methylation in the CpG context, we assumed that ^m^Cp^m^CpG and Cp^m^CpG sites in the *ibm1* mutant are CpCpG sites that also have interior cytosines methylated in WT plants.

These datasets (of first generation *ibm1-1* homozygous mutant plants) were used to produce Figure 5 and Supplementary Figure S8, while the *ibm1* dataset from (9) was used to produce Figures 3 and 4; Supplementary Figures S1, 5 and 6. We also investigated transgenerational effects and confirmed that regions that gain CpHpG methylation in the second generation of *ibm1* mutant (9) completely include the DMBs detected in the first generation of *ibm1* (Supplementary Figure S7D).

### Differentially methylated bins (DMBs)

To compute DMBs we used *DMRcaller* (20), which is an R/Bioconductor package (21,22). Briefly, we considered 100-bp tilling bins and performed a Score test (leading to results similar to a Fisher's exact test) between methylated and total reads in a bin for WT and mutant plants. We selected bins where the *P-*value was less than 0.01, the difference in methylation level was at least 40% in the CG context, 20% in the CHG context or 10% in the CHH context, with at least four cytosines; each cytosine had on average at least four reads, as applied previously in (9).

### ChIP-seq datasets

In our analysis, we used the ChIP-seq datasets for H3 (GSM1242392) and H3K9me2 (GSM1242393) from (10). In addition, we also used ChIP-chip datasets for H3K9me2 in WT (GSM566673) and *ibm1* (GSM566674) published in (23).

### Computational predictions of KYP binding affinity

Three-dimensional models of KYP bound to different DNA sequences were generated using the crystal structure of KYP in complex with ^m^CpHpH DNA and the H3 (1-15) peptide [PDB:4QEO] (6) as a starting model. Changes in the DNA sequence were made using the FOLDX software (24,25). Models were energy minimized and equilibrated using the GROMACS package (26) with the CHARMM36 force fields (27–29). The initial shortest distance between the protein and the box boundaries was set to 1.2 nm. The energy of the system was minimized in vacuum when maximum force on any atom was less than 100 kJ/mol/nm with a maximum of 10 000 steps. The system was then equilibrated with a short MD run of 25 000 steps with a 2-fs time-step (a total of 50 ps). The system was simulated in the NVT ensemble by keeping the temperature (300K) constant; a weak coupling (performed using the Berendsen method) (30) to external heat baths was applied (relaxation times 0.1 ps). Protein and non-protein were coupled to separate baths in order to ensure even distribution of velocities (and therefore temperature) across the system. All covalent bonds were constrained using the LINCS algorithm and non-bonded interactions were computed using the PME method (31) with a grid spacing of 0.12 nm for electrostatic contribution. Interaction energies between KYP and DNA were calculated using the FOLDX software (24,25).

## RESULTS

### Regulatory links between CpG methylation and double methylation at CpCpG sites

**Figure 1.**
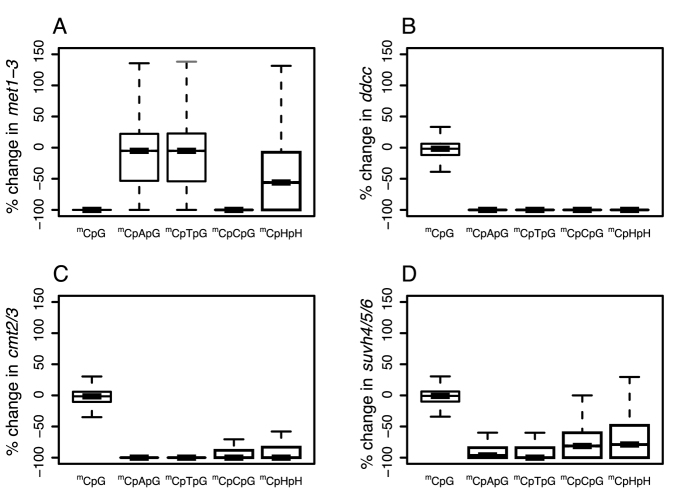
Relative changes in methylation level in four epigenetic mutants (met1-3, ddcc, cmt2/3 and suvh4/5/6) compared to WT. Cytosines in the CpG, CpApG, CpTpG, CpCpG (methylation of the external cytosine) and CpHpH sequences were considered separately (see ‘Materials and Methods’ section).

To examine regulatory links between MET1 and maintenance of methylation of external cytosines at CpCpG sites, we studied methylation patterns in each sequence context (CpG, CpApG, CpCpG, CpTpG and CpHpH) in selected epigenetic mutants of *A. thaliana* and their combinations (*met1-3, ddcc, cmt3, cmt2, cmt2/3, suvh4* also known as *kyp1, suvh4/5/6, drm1/2* and *ibm1*—see ‘Materials and Methods’ section for more information) (9,10) (Figure 1 and Supplementary Figure S1).

Our results confirmed the observation of Yaari *et al.* (15) in the *met1-3* mutant with complete loss of methylation at CpGs that methylation of external cytosines at CpCpG sites is also lost, while cytosine methylation at CpApG and CpTpG sites is not affected (Figure 1A). In addition, complete loss of CpHpG methylation occurs in the quadruple *ddcc* mutant, where DRM1/2 and CMT2/3 are mutated (Figure 1B) (3,10). Thus, while the methylation of CpApGs and CpTpGs depend only on the CMT2/3 pathway (Figure 1A and C), maintenance of methylation of the external cytosine at CpCpGs always requires MET1 (Figure 1A and C) in combination with CMT2/3 or with RdDM pathway, however, the latter contributes to much lower extent (Supplementary Figure S1D).

Although, in the *cmt2/3* double mutant, there is a massive reduction in CpHpG methylation, a low level of methylation of the external cytosines at CpCpGs is still retained (Figure 1C). For the *cmt2/3* double mutant, the loss in CpCpG methylation displays a bimodal distribution, with some CpCpG sites losing methylation completely and others showing residual levels (Supplementary Figure S2A).

Since, the entire CpCpG methylation is erased in the *ddcc* mutant, we concluded that the residual methylation in *cmt2/3* is maintained by the RdDM pathway. We selected CpCpGs for which at least 99% of the WT methylation is lost in the *cmt2/3* double mutant (40 968 sites) as CMT2/3 dependent (CDCs). Alternatively, CpCpG sites in *cmt2/3* that retained more than 20% of the WT methylation level (10 216 sites) at external cytosines were considered as RdDM-dependent sites (RDCs) (Supplementary Figure S2A and B). Indeed, in the *drm1/2* double mutant, RDCs are more affected than CDCs (Supplementary Figure S2C).

Since, methylation of external cytosines of CpCpGs is lost in *met1-3*, we examined in more detail possible links between the change in methylation of these cytosines and the methylation of internal cytosines at CpCpG sites. It became apparent in WT plants, where the internal cytosines at CpCpG sites are unmethylated, that the external cytosines also remain unmodified. In contrast, where the internal cytosines are methylated, the external cytosines are also methylated at ∼40% of CpCpG sites (Supplementary Figure S3). This correlation suggests a regulatory link in which methylation of internal cytosines is necessary but not sufficient for methylation of external cytosines. Thus, in the DNA methylation at CpCpG sites only three of the four possible methylation patterns occur, i.e. CpCpG, Cp^m^CpG and ^m^Cp^m^CpG. Importantly, the fourth option of ^m^CpCpG is almost completely excluded.

**Figure 2.**
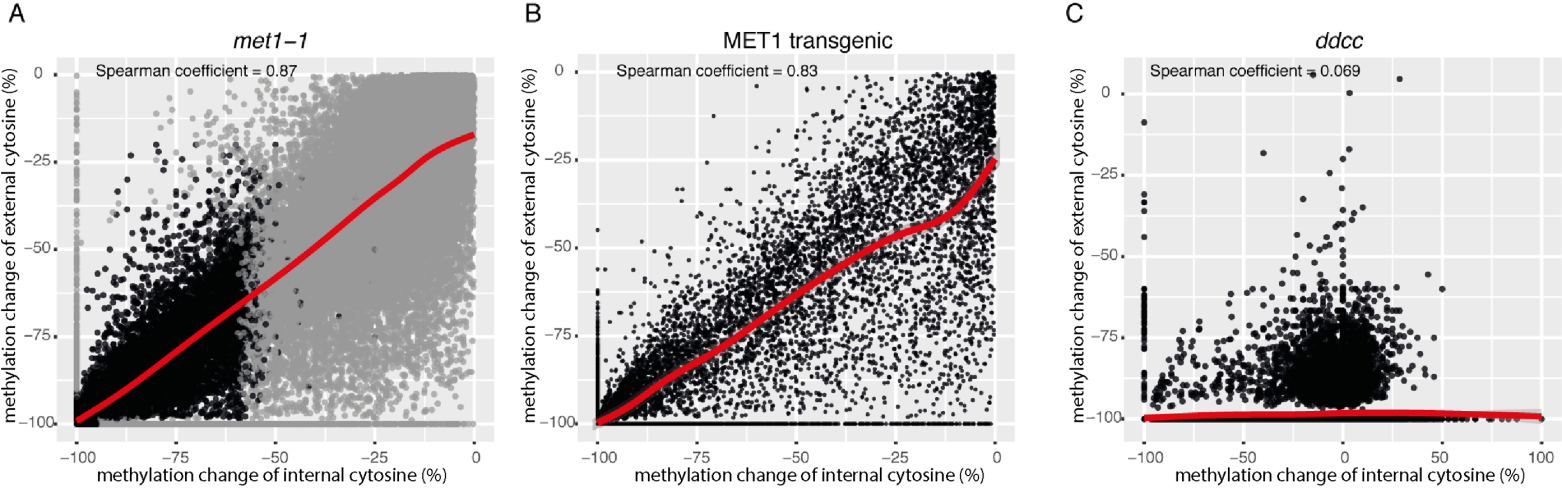
Correlation of internal and external cytosine methylation at CpCpG sites. (**A**) The percentage loss of methylation in the *met1-1* mutant at CpCpG sites displaying at least 50% methylation of both external and internal cytosines in WT plants (64 727 sites). The Spearman correlation coefficient between losses in internal and external cytosine methylation was 0.87. Black points indicate a subset of sites displaying <40% methylation at both external and internal cytosines in the *met1-1* mutant (23 612). (**B**) Changes in methylation levels of external and internal cytosines at CpCpG sites between complemented MET1 transgenic lines and WT plants. Only CpCpG sites displaying at least 50% methylation of both external and internal cytosines in WT plants and less than 40% methylation of external and internal cytosines at CpCpGs in the *met1-1* mutant (23 612 sites; black points from panel A) were considered. The regain of methylation at external and internal cytosines correlated with a Spearman coefficient of 0.83. The bisulfite sequencing datasets consist of pooled reads of two *met1-1* lines independently complemented by a transgenic *MET1*. (**C**) Changes in methylation levels of external and internal cytosines at CpCpG sites in *ddcc* mutant and WT plants (41 498 sites).

To further test this link by which CpG methylation at CpCpGs may influence non-CpG methylation, we examined whether partial loss of methylation at internal cytosines in the CpG context also results in partial loss of methylation of external cytosines at the same CpCpG sites. For this, we analysed bisulfite sequencing datasets of the *met1-1* allele (Catoni *et al.*, bioRxiv: http://biorxiv.org/content/early/2016/06/08/057794), which reduces methylation in CpGs to 25% of the WT (4). We found a positive linear correlation between methylation depletion at internal cytosines and loss of methylation at external cytosines of the same CpCpG sites (Figure 2A). Furthermore, we investigated whether recovery of the methylation of internal cytosines is correlated with regain of methylation at external cytosines, also at the same CpCpG sites. For this, we analyzed bisulfite sequencing datasets of two transgenic *met1-1* lines complemented by the MET1 transgene (Catoni *et al.*, bioRxiv: http://biorxiv.org/content/early/2016/06/08/057794). Also in this case, a positive linear correlation was observed between regain of methylation in internal and external cytosines at CpCpG sites (Figure 2B). As a control, we tested the relationship between methylation of both cytosines in the *ddcc* mutant (Figure 2C). Erasure of external cytosine methylation in *ddcc* had no effect on the methylation levels of internal cytosines.

**Figure 3.**
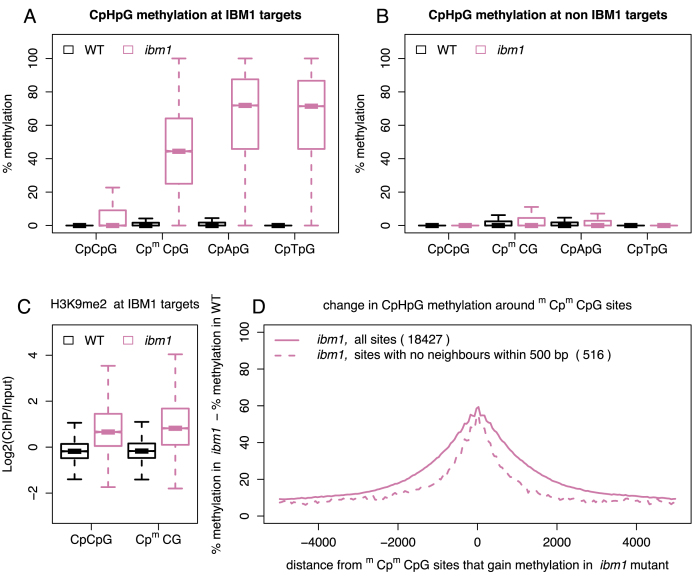
Changes in CpHpG methylation in the ibm1 mutant. (**A** and **B**) show methylation levels of different CpHpG sites (CpCpG, Cp^m^CpG, CpApG and CpTpG) in both WT and *ibm1* plants for IBM1 targets and non IBM1 targets, respectively. (**C**) The levels of H3K9me2 at CpCpG and Cp^m^CpG sites in both WT and *ibm1* plants at IBM1 targets. (**D**) The difference in average CpHpG methylation between *ibm1* and WT plants around ^m^Cp^m^CpG sites that gained methylation in the *ibm1* mutant. Here the ^m^Cp^m^CpG sites (18 427 sites) were defined as having <15% methylation of cytosines in WT and more than 25% methylation in *ibm1* (straight line). We also considered the case of ^m^Cp^m^CpG sites with no neighbours within 500 bp (there are no other ^m^Cp^m^CpG sites within 500 bp) (dashed line). As a control, we also investigated CpG methylation around ^m^Cp^m^CpG sites and our results confirm that there is no change in CpG methylation in the *ibm1* mutant (Supplementary Figure S5).

Remarkably, the methylation of external cytosines at CpCpGs depends on the methylation status of the internal cytosines for only ∼40% of CpCpG sites (Supplementary Figure S3). For the remaining 60%, external cytosines remain unmethylated despite methylation of the internal cytosines. To determine the mechanism of this dual regulation, we examined the methylation status at these sites in genes and also in transposable elements. Expressed genes were methylated exclusively in the CpG context and this methylation was present in the coding regions of genes (gene body-methylation). In contrast, transposable elements displayed methylation in both CpGs and non-CpGs (9,10,32). Consistently, we found CpCpG sites with both cytosines methylated (^m^Cp^m^CpG) exclusively in transposons, promoters of genes regulated by methylation and other loci methylated in all sequence contexts. In contrast, CpCpG sites with only internal cytosines methylated (Cp^m^CpG) were found prevalently in genes but also in a subset of transposons (Supplementary Figure S4A). Loci displaying methylation at both cytosines (^m^Cp^m^CpG) are associated with H3K9me2 levels higher than those when only internal cytosines are modified (Cp^m^CpG) (Supplementary Figure S4B). This relative depletion of H3K9me2 at loci marked by Cp^m^CpG may contribute to the absence of methylation of external cytosines at CpCpG sites, since CMT2 and CMT3 are dependent on H3K9me2 feedback for their DNA methylation activities and, thus, also for the general maintenance of CpHpG methylation (7,10). As a consequence, gene body-methylation, which is restricted to methylation in the CpG context (including the internal cytosines of CpCpGs), seems to be protected against general methylation in the CpHpG context by interruption of the H3K9me2/ CMT2/3 regulatory loop. Histone demethylase IBM1 interferes with this feedback regulation (14). IBM1 targets a subset of genes and removes H3K9me2, thus blocking the spread of methylation in the CpHpG context (14,23). However, protection against non-CpG methylation by IBM1 applies only to a subgroup of body-methylated genes (12%), while the rest of the body-methylated genes are protected against non-CpG methylation also in the absence of IBM1 (Figure 3A and B). The reason for this IBM1 independence is not clear and triggers of the initial acquisition of non-CpG methylation at IBM targets that would promote its subsequent spreading have not been defined.

### The methylation status of CpCpGs influences spreading of CpHpG methylation in body-methylated genes

To investigate possible interdependence of the methylation status at CpCpGs and IBM1activity, we re-analysed the bisulfite sequencing data of WT and *ibm1* mutant plants (9). Genes with Cp^m^CpGs in their bodies gained CpHpG methylation in the *ibm1* mutant, including external cytosines of Cp^m^CpGs (Figure 3A). However, CpCpG sites with unmethylated internal cytosines were only negligibly affected by the *ibm1* mutation (Figure 3A), despite showing a similar gain in H3K9me2 (Figure 3C). We also observed that gain of methylation at CpApGs and CpTpGs decreased with increasing distance from Cp^m^CpG sites, which in the *ibm1* mutant acquire methylation of external cytosines (Figure 3D). There is no change in CpG methylation around Cp^m^CpGs sites (Supplementary Figure S5), which suggests that such an increase in CpHpG methylation is independent of changes in the CpG methylation pattern (Supplementary Figure S5). This finding is consistent with the hypothesis that methylation of the external cytosines at Cp^m^CpGs may be an initial event promoting spreading of CpHpG methylation in gene bodies of the *ibm1* mutant.

**Figure 4.**
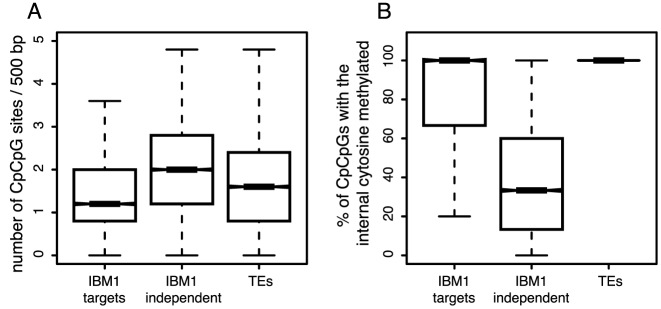
Genetic and epigenetic features of IBM1 targets. Considering 500-bp tilling bins, we defined ‘IBM1 targets’ as bins that display gene body-methylation in WT plants and gained at least 50% methylation in the CpHpG context in the *ibm1* mutant (8815 bins). Bins defined as ‘IBM1 independent’ showed gene body type methylation in WT but did not gain more than 5% methylation in the CpHpG context in the *ibm1* mutant (18 067 bins). Bins defined as TEs had at least 50% methylation in the CpHpG context in WT (14 942 bins). (**A**) The number of CpCpG sites and (**B**) the percentage of CpCpG sites with methylated internal cytosines. To determine whether the three distributions in (A) are different, we performed three pairwise Wilcoxon tests (IBM1 targets compared to IBM1 independent, IBM1 targets compared to TEs and IBM1 independent compared to TEs); in each case *P* < 2.2e-16.

Since a subset of body-methylated genes resist hypermethylation in the CpHpG context even in the *ibm1* mutant, we searched for possible differences between this group and genes that increase in methylation in *ibm1*. We partitioned the genome into 500-bp tiling bins and selected regions displaying gene body-methylation with at least 10% of methylation in the CpG context and less than 5% methylation in the CpHpG or CpHpH contexts. We then selected bins with increased CpHpG methylation of at least 50% in the *ibm1* mutant and named these bins IBM1 targets, while bins with an increase in CpHpG methylation of <5% where considered to be IBM1 independent. Although IBM1 targets had on average fewer CpCpG sites (Figure 4A), nearly all were methylated in the internal cytosines (Figure 4B). In contrast, most CpCpG sites in IBM1-independent regions showed unmethylated internal cytosines (Figure 4B). These observations are consistent with the hypothesis that IBM1 targets gain methylation in the *ibm1* mutant by CpHpG methylation initiated by modification of external cytosines at Cp^m^CpGs. Therefore, the presence/absence of Cp^m^CpGs can be used to predict IBM1 targets in contrast to other CpG sites (Supplementary Figure S6).

IBM1 gene expression is completely suppressed in the *met1-3* mutant due to depletion of DNA methylation in a region encoding the *IBM1* intron (33). Notably, we observed only a small overlap between regions that gain CpHpG methylation in *met1-3* and in *ibm1* (Supplementary Figure S7A). Since in *met1-3* Cp^m^CpGs become CpCpGs, they are not targeted by CMT3. Moreover, if CpHpG methylation is initiated independently of Cp^m^CpG sites, we should detect an increase in CpHpG methylation in *met1-3*, being an *ibm1* epi-mutant, at genes that are IBM1 targets. However, CpHpG increase of methylation in *met1-3* mutant occurs mostly at TEs (Supplementary Figure S7B) and CpApG/CpTpG sites did not gain methylation at IBM1 targets (Supplementary Figure S7C), which is consistent with the hypothesis that Cp^m^CpGs are indeed required for the initiation of methylation in the CpHpG context at IBM1 targets in the absence of IBM1 activity.

**Figure 5.**
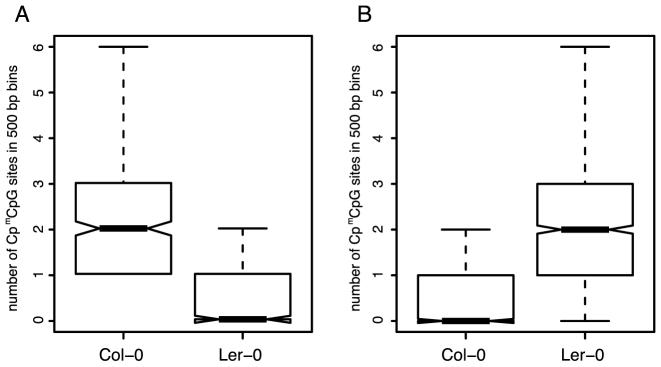
The effect of inter-ecotype variation in Cp^m^CpGs on the capacity of a region to gain methylation in the ibm1 mutant. Boxplot of the number of Cp^m^CpG sites in Col-0 and Ler-0 for homologous 500-bp bins that gained CpHpG methylation exclusively in (**A**) Columbia- Col-0 or (**B**) Landsberg (Ler-0).

Finally, to further investigate the activity of Cp^m^CpG sites as coordinators of CpHpG methylation, we generated bisulfite sequencing datasets for the first generation of *ibm1* homozygous mutant in Columbia (Col-0) and Landsberg (Ler-0) ecotypes in which the same *ibm1* mutant allele was introgressed. We analyzed 500-bp regions in Col-0 that displayed gene body-methylation and also had homologous sequences in Ler-0. From this set, we selected bins displaying at least 50% CpHpG methylation in Col-0 (500 bins) or Ler-0 (1337 bins). Only 87 bins showed increased CpHpG methylation in both ecotypes; the majority of the bins displayed an increase in only one ecotype (Supplementary Figure S8). Interestingly, bins that gained CpHpG methylation only in the Col-0 ecotype (413 bins with CpHpG methylation of at least 50% in Col-0 and <5% in Ler-0) had CpCpG sites with internal cytosines methylated in Col-0 (on average 2) and not in Ler-0 (on average zero) (Figure 5A). In contrast, bins that gained CpHpG methylation only in the Ler-0 ecotype (1250 bins with CpHpG methylation of at least 50% in Ler-0 and <5% in Col-0) had CpCpG sites with internal cytosine methylation in Ler-0 (on average 2) and not in Col-0 (on average zero) (Figure 5B). These results provide additional support for the conclusion that the presence of Cp^m^CpG sites promotes CpHpG methylation and that regions gain CpHpG methylation in the *ibm1* mutant only when they include these particular sites.

Possible mechanisms by which CpHpG methylation is initiated at Cp^m^CpGs are: (i) CMT2/3 first methylate the external cytosines of Cp^m^CpG sites and KYP then recognizes ^m^Cp^m^CpGs and adds H3K9me2 marks, or (ii) KYP binds directly to Cp^m^CpGs and the added H3K9me2 marks start a self-reinforcing loop with CMT2/3. To test the likelihoods of these two possibilities, we examined the crystal structure of KYP bound to DNA (6) and computed the binding energies of KYP to various DNA sequences using molecular dynamics simulations. Since KYP displayed the stronger binding affinity when both cytosines are methylated (^m^Cp^m^CpG, Supplementary Figure S9) than binding to Cp^m^CpGs, it could be hypothesized that CMT2/3 may need to methylate the external cytosine of Cp^m^CpG sites first and then KYP recognizes and binds ^m^Cp^m^CpGs. Obviously, this hypothesis needs future tests by additional experiments assaying *in vitro* binding and biochemical activities.

## DISCUSSION

Transposable elements display dense and complex DNA methylation patterns with cytosines methylated in CpG, CpHpG and CpHpH sequence contexts. This methylation is established and maintained simultaneously by several DNA methyltransferases involved in distinct methylation pathways. Methylation at CpGs is maintained by MET1, at CpHpGs by CMT2/3 cooperating with KYP and at CpHpHs by DRM2 and CMT2, the former acting in the RdDM pathway (3,8). It is therefore very challenging to dissect regulatory interactions between these pathways at transposon loci, which have complex methylation patterns that vary greatly between different transposons. In contrast, a subset of genes acquires DNA methylation in their bodies, which in WT plants is restricted to CpG sites and maintained by MET1. However, in plants deficient in H3K9 demethylase (IBM1) numerous body-methylated genes gain methylation outside CpGs, predominantly in the CpHpG sequence context, but this does not occur at all body-methylated genes. Therefore, such genes can be classified as either targets of IBM1 or non-targets of IBM1. The latter seem to be protected against invasion of ectopic non-CpG methylation in an IBM1-independent manner. The factors contributing to this protection were unknown.

Here, we provide evidence that those body-methylated genes that do not gain CpHpG methylation in the *ibm1* mutant contain CpCpG sites free of methyl groups (Figures 3–5). In contrast, genes with CpCpGs having methylated internal cytosines acquire CpHpG methylation in the absence of IBM1 protective activity.

Previous work (7) suggested that CMT3 can *de novo* methylate CpHpG sites. In addition, it has been shown that KYP binds weakly to ^m^CpG sites when these are flanked by adenines (A^m^CpGA) (11). KYP binding occurs through the SRA domain and the flipped-out methylated cytosine (6). Therefore, KYP could bind to Cp^m^CpGs (recognising the methylated internal cytosines) or to ^m^Cp^m^CpGs (recognising either the methylated internal or external cytosines). Our structure simulation data favor the second possibility (Supplementary Figure S9); however, additional studies of KYP binding specificities are necessary to further test this hypothesis.

Nevertheless, such a scenario is compatible with the model proposed by Yaari *et al.* (15) where ^m^CpCpG methylation mediated by CMT3 depends on the methylation of the second strand of DNA at ^m^CpGpG, which is mediated by MET1. Based on the observation that the external cytosine methylation at CpCpG decreases in *met1* mutants of *Physcomitrella patens* and *Arabidopsis* they proposed that CMT3 is unable to methylate the symmetric CpGpG site, where methylation is maintained exclusively by MET1 (15). However, their model also considers propagation of methylation at CpCpG sites in which the internal cytosine is unmethylated, a situation that is excluded in *Arabidopsis* (Supplementary Figure S3). Therefore, pre-existing body methylation and CpCpG sites with methylated internal cytosine are both needed for directing CpHpG methylation in *Arabidopsis* genes. Our results suggest that IBM1 prevents increase of ectopic non-CpG methylation in these genes, most likely by preventing its spreading from the Cp^m^CpG sites (Figure 3D). This assumption does not require IBM1 to be targeted to a specific subset of genes. IBM1 may simply be available at all genic regions and be activated only when *de novo* CpHpG methylation is initiated by double methylation at Cp^m^CpG sites and the appearance of H3K9me2. On the other hand, it is known that DNA mutation rates are influenced by DNA methylation (34) and genomes containing methylated cytosines tend to become depleted in CpG sites (35). Our findings imply mutation constraint of CpCpG sites; mutation of these sequences could influence the epigenetic landscape of body-methylated genes. As a matter of fact, the CpG dinucleotide is avoided in codon usage in *Arabidopsis* and other plants (36).

A possible link between CpG and CpHpG methylation was recently proposed by Bewick *et al.* (37), who reported that the absence of CMT3 in *Eutrema salsugineum* and *Conringia planisiliqua* results in the absence of gene body-methylation. It was proposed that stochastic establishment of CpHpG methylation followed by its stochastic removal can lead gradually to the establishment of gene body-methylation only in the CpG context. As a consequence, the absence of CMT3 during the evolution of these species has resulted in erasure of methylation in gene bodies (37). Our data complement the proposed evolutionary link between CpG and CpHpG methylation by providing further evidence of a regulatory relationship between CpG and CpHpG operating in *Arabidopsis*.

A direct influence of CpG methylation on the development of further epigenetic properties of loci is likely to be of crucial importance in the maintenance and inheritance of these properties through mitosis and meiosis. MET1-mediated inheritance of CpG methylation patterns through DNA replication cycles is essential for epigenetic identity, as evidenced by the transgenerational persistence of epigenetic deficiencies triggered by short-term depletion of MET1 (38). In contrast, epigenetic alterations resulting from depletion of factors involved in non-CpG methylation are not transgenerationally inherited. We propose here that CpCpG sites may act as a scaffold for crosstalk between CpG and non-CpG methylation pathways, constituting a possible mechanism by which CpG methylation may maintain locus epigenetic identity in the absence of non-CpG methylation. Thus, our results define CpG methylation as a crucial epigenetic mark providing broader epigenetic identity and the means for its stable inheritance.

## ACCESSION NUMBER

Sequencing data have been deposited in Gene Expression Omnibus under the accession number GSE89913.

## Supplementary Material

Supplementary Data
