# Supplementary material for: Cytosine methylation at CpCpG sites triggers accumulation of non-CpG methylation in gene bodies
Source: Nucleic Acids Res. 2017 Jan 4;45(7):3777–84. doi: 10.1093/nar/gkw1330 (PMC5397141; doi:10.1093/nar/gkw1330)
Supplement: Supplementary Data [file gkw1330_Supplementary_Data.zip › nar-03086-v-2016-File007.pdf]

Supporting Information

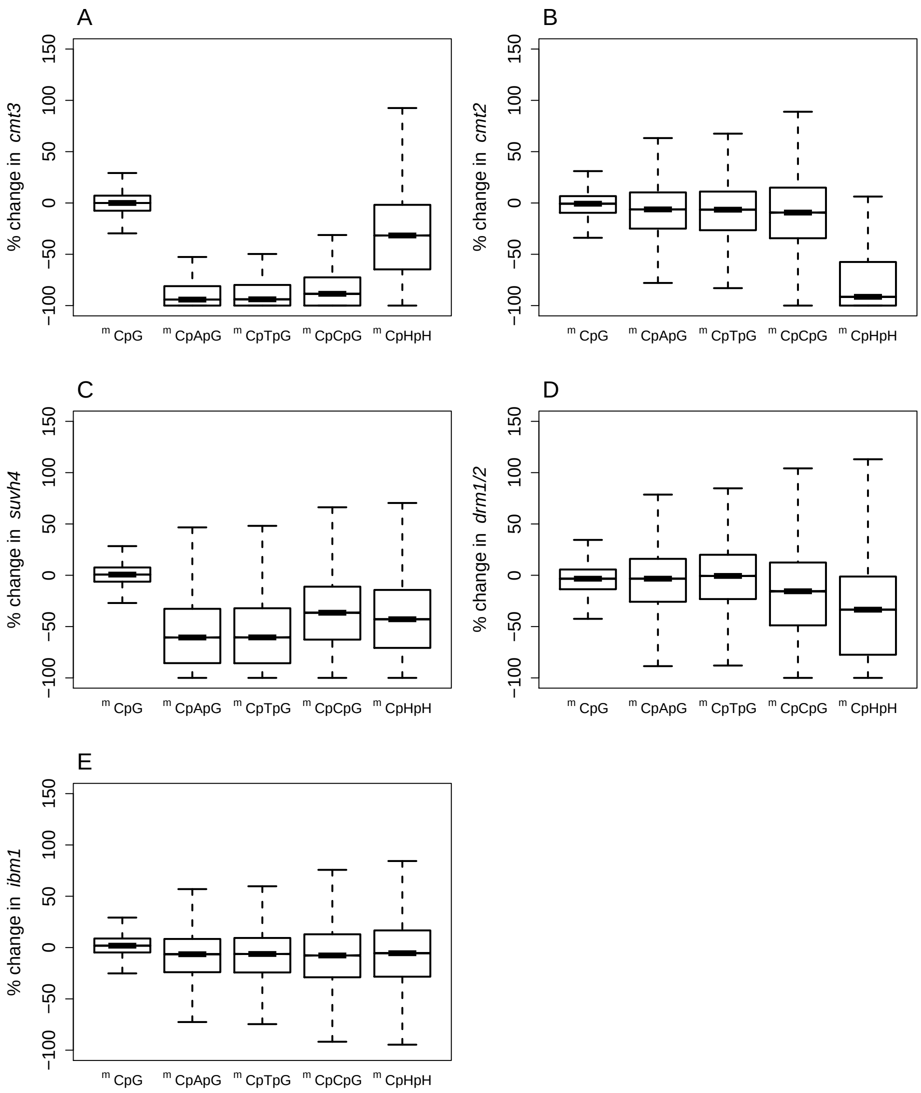

Figure S1: **Relative changes in methylation levels in five epigenetic mutants (*cmt3*, *cmt2*, *suvh4*, *drm1/2* and *ibm1*) compared to WT.** We considered separately cytosines in the CpG, CpApG, CpTpG, CpCpG (methylation of the external cytosine) and CpHpH sequences (see *Materials and Methods*).

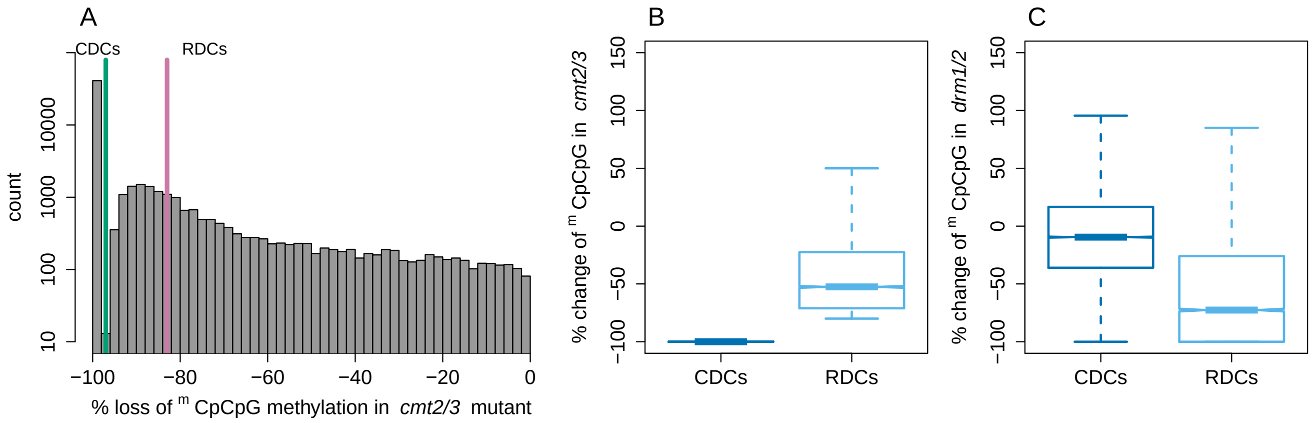

Figure S2: **Major mechanisms involved in the maintenance of methylation at CpCpG sites.** (A) The distribution of changes in ^{m}CpCpG in the *cmt2/cmt3* double mutant. The majority of the cytosines completely lost ^{m}CpCpG, while a small portion appeared to retain some methylation. CpCpG sites that lost at least 99% methylation were classified as CDCs and sites that lost less than 80% methylation as RDCs. (B, C) Relative changes in methylation levels of CDCs and RDCs in the *cmt2/3* double (B) and the *drm1/2* double mutant (C).

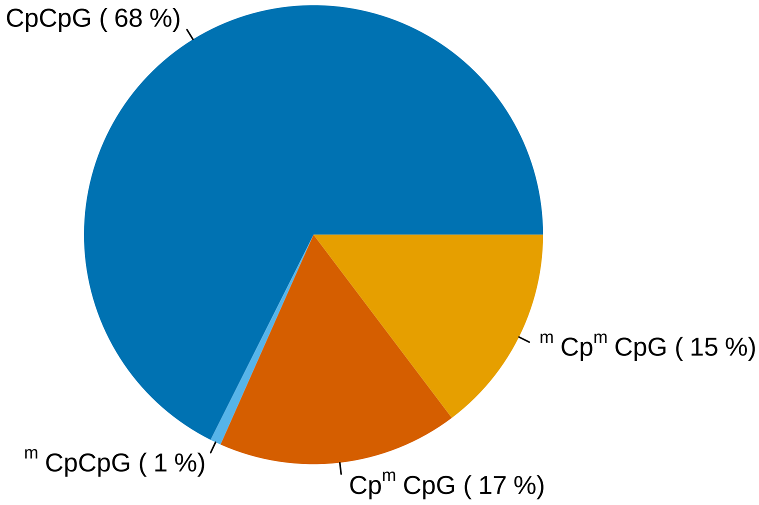

Figure S3: **Relationship between methylation in CpG and CpCpG contexts.** Based on methylation of external and internal cytosines, CpCpG sites were classified as: CpCpG, ^{m}CpCpG, Cp^{m}CpG, or ^{m}Cp^{m}CpG. External cytosines (in the CpHpG context) were considered methylated if the methylation level was at least 15%; internal cytosines (in the CpG context) were considered methylated if the methylation level was at least 30%.

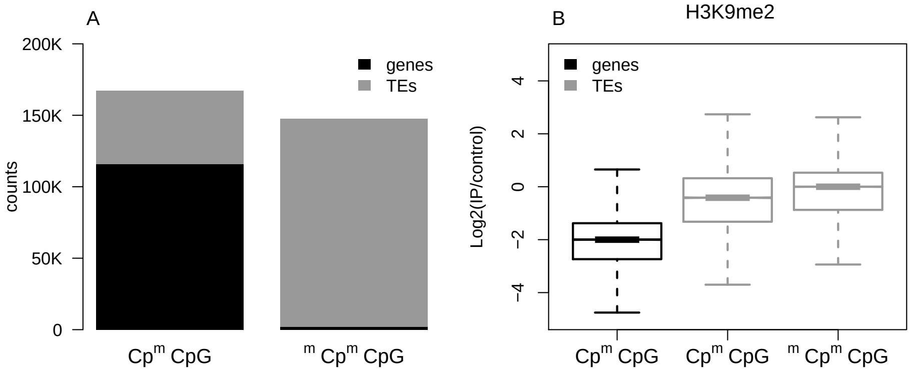

Figure S4: **Distribution of CpCpG sites with both cytosines methylated or only the internal cytosine methylated.** (A) Number of Cp^{m}CpGs and ^{m}Cp^{m}CpGs within genes (methylated exclusively at CpGs) or loci methylated in all sequence contexts, mostly transposons (TEs). From the methylation data in the three contexts in 500-bp tiles, bins with more than 10% ^{m}CpG methylation and less than 5% ^{m}CpHpG or ^{m}CpHpH methylation were classified as genes. Bins with at least 10% ^{m}CpG methylation and at least 5% ^{m}CpHpG or ^{m}CpHpH methylation were selected as TEs. (B) H3K9me2 ChIP-seq data from (10) for Cp^{m}CpG and ^{m}Cp^{m}CpG sites. For Cp^{m}CpG sites, we considered separately the case of genes and TEs. The three distributions were significantly different (Wilcoxon test; $P < 2.2e\text{-}16$).

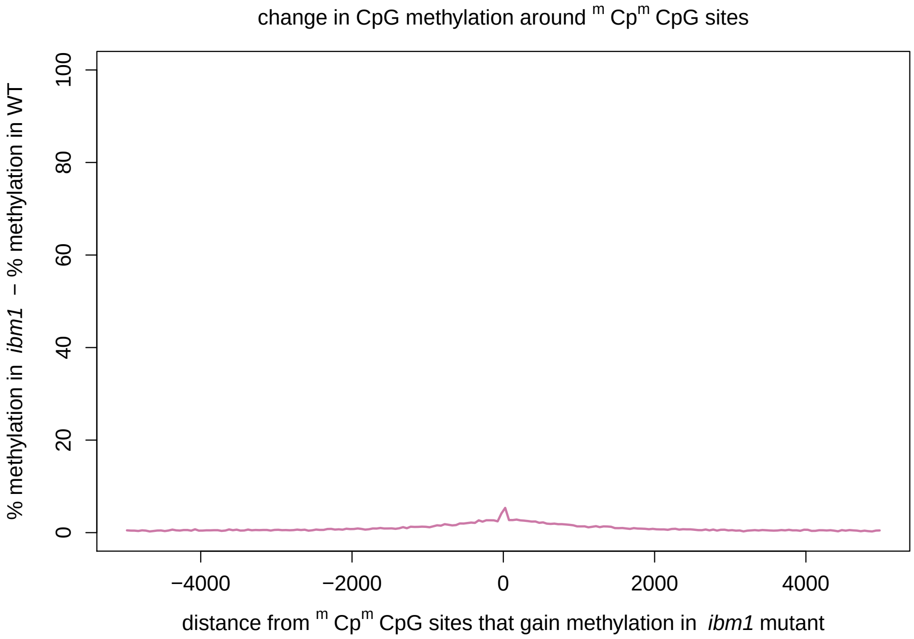

Figure S5: **Change in CpG methylation around ^{m}Cp^{m}CpG sites in gene bodies that gained methylation in *ibm1* mutant.** We plot the difference in CpG methylation between *ibm1* mutant and WT plants around ^{m}Cp^{m}CpG sites where the external cytosine was unmethylated in WT (displayed less than 15% methylation) and was methylated in *ibm1* mutant (displayed more than 25% methylation). The average was computed over $18,275$ ^{m}Cp^{m}CpG sites in gene bodies using 50 *bp* bins.

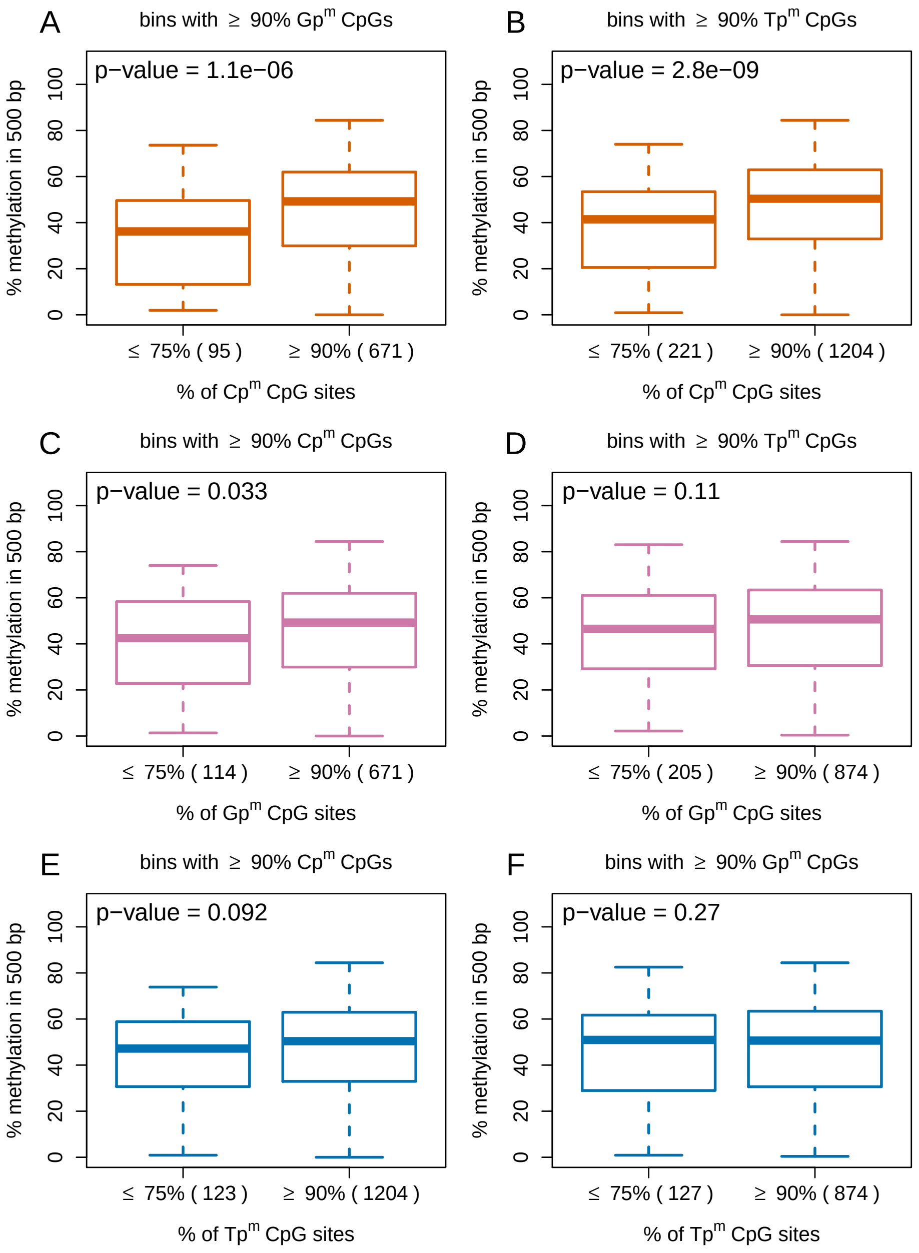

Figure S6: **CpHpG methylation at bodies of genes in *ibm1* mutant.** We selected 3 sets of body methylated 500 bp bins with at least 5 CpG sites ($\geq 90\%$ methylated): in GpCpG, CpCpG and TpCpG context (marked above the plots). Each of the set was split in two subgroups for each particular sequence context (marked below the plots): (i) bins with at least 5 sites and $\leq 75\%$ of the sites methylated and (ii) bins with at least 5 sites and $\geq 90\%$ of them methylated. (A) GpCpG and (B) TpCpG split by CpCpGs, (C) CpCpG and (D) TpCpG split by GpCpGs, (E) CpCpG and (F) GpCpG split by TpCpGs. ApCpG sequences were not included in these analyses due to their inherent low methylation level (mostly $< 70\%$). Wilcoxon Rank Sum test was used to compute the p-values. Initial gene body methylated bins were in wild type $\geq 70\%$ of CpG methylated, had $< 5\%$ non-CpG methylation and contained at least 10 CpGs with $\geq 30\%$ methylation.

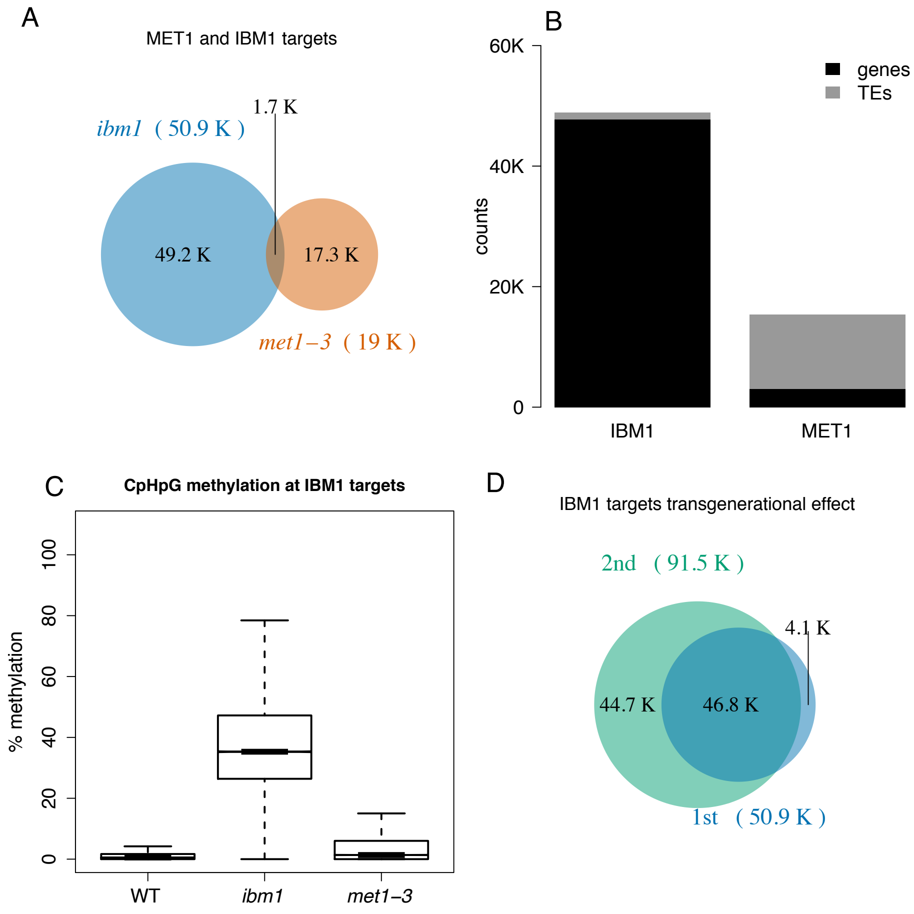

Figure S7: **Definition of IBM1 and MET1 targets.** We computed DMBs (Differentially Methylated Bins) between mutant (first generation *ibm1* and *met1-3*) and WT plants and defined MET1 targets as CpHpG hypermethylated DMBs in *met1-3*, where CpHpG methylation increased in the *met1-3* mutant relative to WT plants; see *Materials and Methods*. Similarly, we defined IBM1 targets as CpHpG hypermethylated DMBs in *ibm1*, where CpHpG methylation increased in the *ibm1* mutant relative to WT plants. (A) Overlap between IBM1 targets and MET1 targets. (B) The number of IBM1 targets and MET1 targets in genes and TEs; see Figure S4 for definitions of genes and TEs. (C) CpHpG methylation level at IBM1 targets. (D) The overlap between the IBM1 targets in first generation *ibm1* mutant (the dataset generate as part of this work) and second generation *ibm1* mutant, data from reference (9).

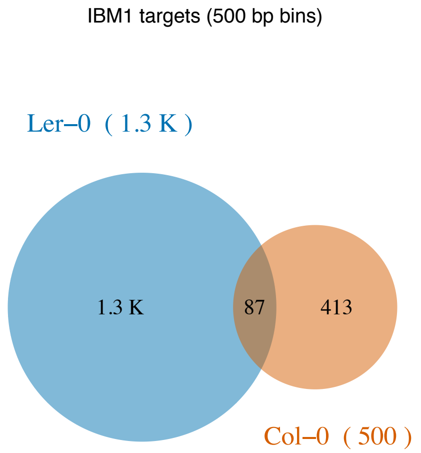

Figure S8: **Overlap between IBM1 targets in Col-0 and Ler-0.** We considered all 500-bp bins that displayed gene body methylation in Col-0 with a homologous sequence in Ler-0. We then selected bins showing at least 50% CpHpG methylation in ibm1 in the Col-0 (red) and Ler-0 backgrounds (blue).

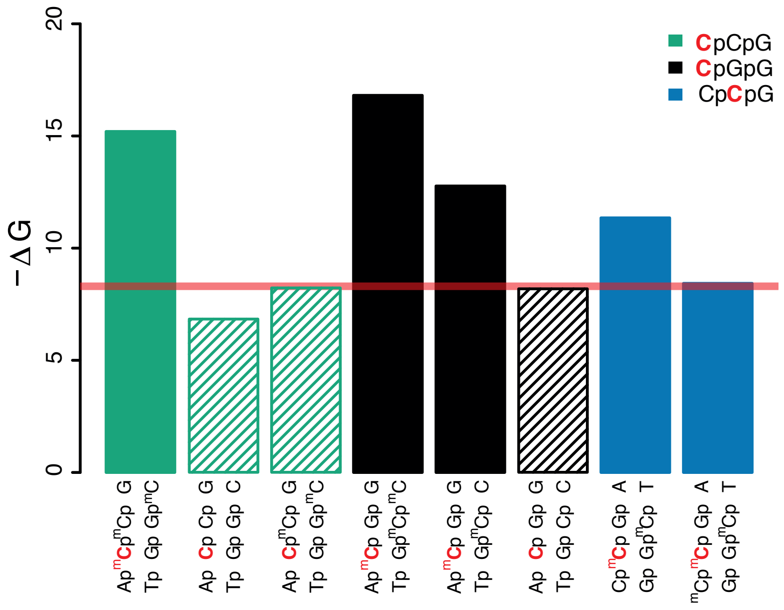

Figure S9: **Binding energy of KYP to different DNA sequences.** Filled bars resent cases where KYP can recognises a flipped methylated cytosine and dashed bars when the cytosine that is in contact with KYP is not methylated (marked in red bold). The horizontal red line separates the case of non-specific binding of KYP to unmethylated Cs from the case of specific binding of KYP to methylated Cs. CpCpG sites have three cytosines and we considered that KYP can recognise each of the three cytosines: the external one on the positive strand (green bars), the internal one on the positive strand (blue bars) and the one on the negative strand (black bars).

Table S1: **Sequencing and methylation call**.

**Sample**	**Total reads**	**Trimmed**	**% kept**	**mapping**	**Deduplicated**	**Total kept reads**	**Plastide conversion**	**CpG**	**CpHpG**	**CpHpH**
ibm1 Ler-0	26,023,931	24,463,641	94.00%	66.60%	82.06%	13,365,540	0.9857	44.60%	27.20%	5.80%
ibm1 Col-0	31,607,751	29,973,205	94.83%	66.20%	78.53%	15,573,853	0.9866	41.30%	20.90%	5.50%
